# Assessment of Proliferation and Apoptosis in Testes of Rats after Experimental Localized Electron Irradiation

**DOI:** 10.3390/cimb44110391

**Published:** 2022-11-18

**Authors:** Grigory Demyashkin, Sergey Koryakin, Aleksandr Moiseev, Vyatcheslav Saburov, Margarita Zatsepina, Maya Epifanova, Yulia Stepanova, Vladimir Shchekin, Matvey Vadyukhin, Petr Shegay, Andrei Kaprin

**Affiliations:** 1 National Medical Research Radiological Centre of the Ministry of Health of the Russian Federation, Sechenov University, 249036 Obninsk, Russia; 2 Institute of Clinical Medicine, I. M. Sechenov First Moscow State Medical University (Sechenov University), 119991 Moscow, Russia; 3 Department of Urology and Operative Nephrology, Peoples’ Friendship University of Russia (RUDN University), 117198 Moscow, Russia

**Keywords:** electron irradiation, male infertility, spermatogenesis, seminal balls

## Abstract

Background and purpose: With the emergence of linear accelerators in radiotherapy, it becomes necessary to accurately select new dosing regimens. The purpose of this study was to assess the morphological changes of spermatogenesis after radiation exposure. Materials and methods: Male Wistar rats (n = 40) were subjected to targeted ionizing radiation on a pulsed electron accelerator “NOVAC-11” with doses of 2, 8 and 12 Gy. Spermatogenesis was assessed a week later using light microscopy and immunohistochemical method (antibodies to Ki-67, Bcl-2, p53, Caspase 3). Results: A decrease in the number of normal germ cells was seen in all experimental groups, while radioresistant Sertoli and Leydig cells were barely affected. The most serious damage to the tubules and germ cells was observed in 8 and 12 Gy irradiation groups. IHC analysis of testes after irradiation showed a shift in the proliferative-apoptotic balance toward apoptosis of germ cells: a decrease in the expression levels of Ki-67 and Bcl-2, an increase in p53-positive and caspase 3-positive cells by the end of the experiment. Conclusion: Dose-dependent progressive pathomorphological changes in histoarchitectonics of the testes are traced, and a decrease in the number of germ cells is seen on the seventh day after irradiation with a pulsed electron accelerator “NOVAC-11”.

## 1. Introduction

Infertility is a major health problem worldwide and is estimated to affect 8–12% of couples in the reproductive age group. A Global Burden of Disease survey reported that between 1990 and 2017, the age-standardized prevalence of infertility increased annually by 0.370% in women and by 0.291% in men. The cause of infertility lies solely with the man in 20–30% of cases, and a male cause is contributory in a further 20% [1]. Various etiologies of this disease, the complexity of the pathogenesis, as well as the functional interaction of the testes with other systems and organs, create great difficulties in the development of adequate treatment methods.

Radiation exposure plays a crucial role in spermatogenesis disorders, along with such risk factors as bad habits, the influence of heat, chemicals, anatomical features, and hormonal misbalance. People who live in zones of radionuclide pollution and near nuclear facilities may endure the consequences of past man-made disasters [1].

Radiation therapy is successfully used in medical practice for malignancies treatment. In particular, reproductive system malignancies are the leading cause in the structure of oncological mortality [2].

For general or targeted effects in external beam radiotherapy, there can be used cobalt machines that emit high activity γ-rays, linear electron accelerators, which produce both high-energy and direct electron beam (β-irradiation), and other machines with ionic, proton and neutron types of irradiation. The advantages of targeted radiotherapy are the minimal damage risk to neighboring organs under the maximum possible radiation dose, high rates of effectiveness and favorable prognosis for patients.

Nevertheless, with the emergence of new diagnostic and therapeutic methods of radiology, we face the problem of their safety. The selection of optimal radiation doses, their proper adjustment and leveling of their toxicity are the key points that have become an increasingly important task of modern oncology.

The effect of radiotherapy on the human reproductive system is a controversial issue. It is known that radiotherapy is characterized by a gonadotoxic impact on the male organism and significantly reduces the number of dividing spermatogonia and spermatocytes and can lead to azoospermia [3]. Disorder of spermatogenesis occurs at a low radiation dose of 0.1–1.2 Gy, and irreversible testes damage occurs at a dose of 4 Gy, though spermatogenesis recovery can also be possible [4].

Recent research results report the more aggressive impact of targeted γ-ray irradiation compared to the general one; however, it is important to consider, for example, electron beam therapy, which is widely used nowadays because of its high success rate in the treatment of, for example, gynecological malignancies [5]. At the same time, the effects of this kind of radiation on spermatogenesis remain unexplored, and this raises a number of questions: whether the apoptotic rate of male gametes is accelerated, on which stages of spermatogenesis they are more likely to be damaged, and for how long these pathomorphological changes persist after electron irradiation.

γ-Rays are used in most of the existing experimental models of male infertility. Because of the appearance of the linear electron accelerators, there is a need for a precise selection of new dosing regimens and detailed studying of this perspective method. Hence, in our study, for the first time, we highlight the use of local electron irradiation on testes and estimate its effects on rats’ spermatogenesis.

The objective of the study: The assessment of proliferation and apoptosis in testes of rats after local electron irradiation under different dosing regimens (experimental research).

## 2. Materials and Methods

The experimental study was carried out on the basis of the Medical Radiological Scientific Center, named after A.F. Tsyb.

### 2.1. Animal Model for In Vivo Study

Male Wistar rats (220 ± 20 g; 9–10 weeks; n = 40) were kept in a vivarium, two animals in each plastic cage under a controlled temperature (22 °C), with a 12 h light day, air conditioning at a temperature of 23 °C and humidity 40–60%, on a standard diet with water ad libitum. Rats were randomly divided into control (I; n = 10) and experimental (II–IV; n = 10 in each) groups depending on the dose of single targeted electron irradiation they were exposed to: II–2 Gy, III–8 Gy, IV–12 Gy. The rats were exposed to local abdominopelvic irradiation in the testes projection.

Irradiation procedures were carried out in the Department of Radiation Biophysics of A. Tsyb Medical Radiological Research Center on the pulsed electron accelerator “NOVAC-11” (S.I.T. Sordina Iort Technologies S.P.A., Vicenza, Italy)—the first IOeRT mobile electron linear accelerator designed worldwide in 1997. It is the first example of a radiotherapy device that is flexible and easy to use, powerful and safe. It is able to perform the IOeRT treatment in only 100 s. NOVAC 11 ensures proper coverage of any anatomic region where the IOeRT treatment needs to be delivered. By selecting the correct applicator/energy combination, it is possible to treat any neoplastic disease effectively and safely. NOVAC 11 maximum energy is 10 MeV which allows treating targets with a thickness up to 2.6 cm inside the 90% isodose (3.0 cm inside the 80% isodose). According to the performed dosimetric studies, an electron beam with 10 MeV energy potential guarantees irradiation of the zone of interest with the required dose. Before irradiation, rats of experimental groups were treated with a single administration of ketamine (50 mg/kg, intramuscularly) and xylazine (5 mg/kg, intraperitoneally). Anesthetized rats were placed on the object table one by one, in the supine position, with splayed legs, so that the radiation beam focused on the testes while the lungs and heart remained in the area of radiation shadow. The tube was applied to the irradiated region so that its end was not more than two millimeters above the skin in the perpendicular position to it.

### 2.2. The Removal of Animals from the Experiment

Animals of all groups (I–IV) were removed from the experiment by administration of high doses of anesthetic. After routine euthanasia, the testes were removed from the rats according to the design of the experiment. At the same time, the appearance was evaluated: weight (absolute—in grams and relative—in relation to body weight, in %), size and condition of the parenchyma on the section. Time of death: for all groups, after seven days.

The study was performed in accordance with the “International Recommendations for Biomedical Research Using Animals” (EEC, Strasbourg, France, 1985), the “European Convention for the Protection of Vertebrate Animals used for Experiments or Other Scientific Purposes” (EEC, Strasbourg, France, 1986) and Guidelines for the conduct of biomedical research on the care and use of laboratory animals (ILAR, DELS) and the Rules of laboratory practice and the order of the Ministry of Health of the Russian Federation No. 199n dated 01.04.2016 “On the approval of the rules of laboratory practice”, as well as approved by the Local Ethics Committee of Sechenov University (Protocol No. 043; 11 August 2020).

### 2.3. Morphological Evaluation

After extraction, the appearance of the testes and the state of the parenchyma on the cut were assessed and weighed (in grams). Then they were cut parallel to the sagittal plane every 2 mm, fixed in Bouin’s solution; after insertion (apparatus for histological tissue guiding, Leica Biosystems, Wetzlar, Germany), they were embedded in paraffin blocks, from which serial sections (2 µm thick) were prepared, dewaxed, dehydrated and stained with hematoxylin and eosin (H&E) for histological evaluation.

**Figure 1:**
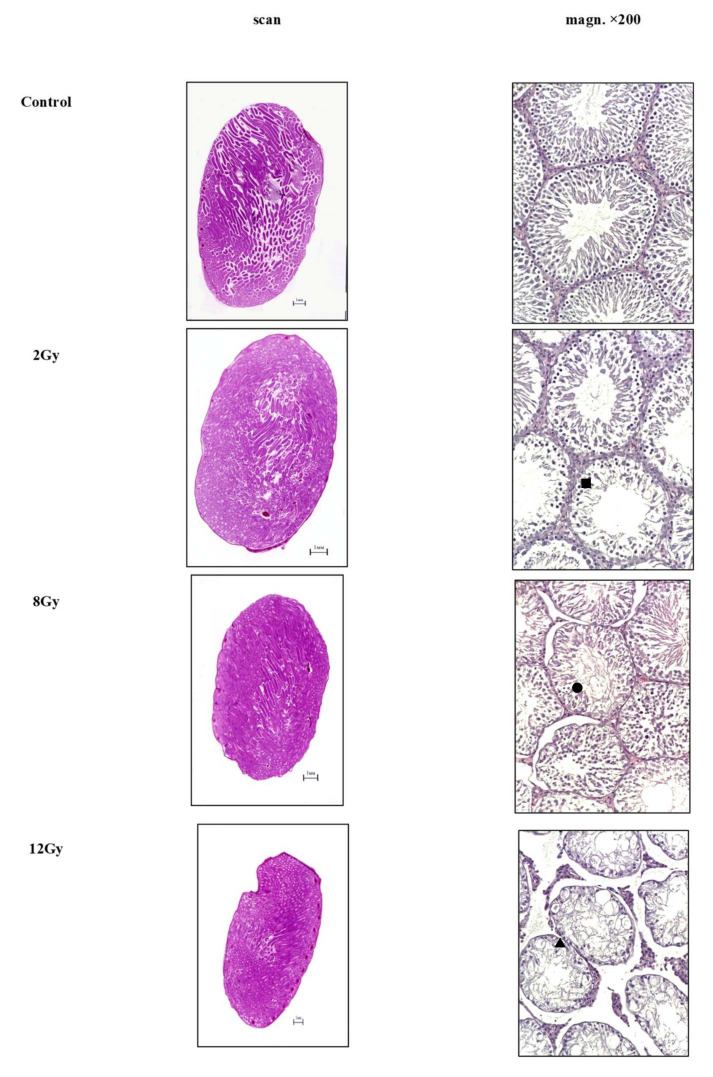
Testes of rats of experimental groups at different radiation doses; stain—hematoxylin and eosin, magn. ×200 (■—the proportion of damaged seminiferous tubules (area—28,079.84 μm^2^; diameter—189.083 μm; *p* < 0.01) with disorganization of the epithelium and loss of cell polarity accounted for up to 1/8 of the testis (1–2 transverse sections of tubules in sight); ●—1/3 of the seminiferous tubules showed the appearance of highly degenerated spermatids and spermatocytes combined into seminal balls (area—0.7 µm^2^; diameter—0.9 µm; *p* < 0.01). The area of single primary spermatocytes was 0.8 µm^2^ at *p* < 0.01, and the diameter was 0.9 µm at *p* < 0.01, which is 1.1 times more than normal; ▲—the largest vacuoles were found in the seminiferous tubules of the irradiation group with a dose of 12 Gy. The number of seminal balls doubled, and their size was 5.0–10.0 times larger than the area of the primary spermatocyte. The dimensions of the aplastic seminiferous tubules are significantly smaller (area—14,123.72 µm^2^; diameter—76.142 µm; height of the spermatogenic epithelium—14.2 ± 12.1 at *p* < 0.01; an increase in the number of pathological mitoses) than in the previous groups, and therefore, an increase in the interstitial component was observed testes with edema. Damage to the seminiferous tubules was 3/4).

Morphological and morphometric analysis was carried out in 10 randomly selected fields of view of the microscope at ×100 and ×200 magnification in 4 random sections from each sample, moving the slides at equal intervals along the X and Y axes, using a semi-automatic image analyzer. Light microscopy was performed using a video microscopy system (Leica DM3000 microscope, Leica Biosystems, Wetzlar, Germany; DFC450 C camera; Platrun LG computer), and morphometric data were obtained using software for image processing and analysis (Leica Application Suite (LAS) Version 4.9.0) (Figure 1). The spermatogenic cycle of rats, which includes 14 consecutive stages, was assessed according to Chermont, Leblond and Messier (1959). Testicular assessment was carried out according to S. Johnsen criteria modified by De Kretser and A. Holstein (Table 1).

### 2.4. Statistical Analysis

The data were statistically processed using SPSS Statistics version 10.0 for Windows (IBM Analytics 11.1, Armonk, NY, USA). The arithmetic mean and the root-mean-square deviation were then calculated. The fact that the results corresponded to the normal distribution was proved using the Kolmogorov–Smirnov criterion. To compare the two samples, t-criterion was used with significance level of *p* < 0.01. In the absence of normal distribution of data, nonparametric F. Wilcoxon (Statistical Methods for Research Workers) criterion was used with significance level of *p* < 0.01.

### 2.5. Immunohistochemical Analysis (IHC)

Immunohistochemical study was performed on paraffin sections according to a standard protocol in an automatic mode in a Ventana immunohistostiner (Roche, Benchmark Ultra, Basel, Switzerland). We used primary antibodies to Ki-67 (MM1, Cell Marque; RTU, Wetzlar, Germany), Bcl-2 (bcl-2/100/D5 Bond; RTU, Wetzlar, Germany), p53 (DO-7, Millipore; 1:200, Burlington, MA, USA) and Caspase 3 (E87, Abcam; 1:300; RTU, Wetzlar, Germany). For each marker, control studies were performed in order to exclude pseudo-positive and pseudo-negative results. Cell nuclei were counterstained with Mayer’s hematoxylin; the sections were washed under running water, dehydrated and embedded in a balm.

Evaluation of the results of immunohistochemical reactions was carried out according to the distribution of staining and using a 3-point system with calculation of the number of immunopositive cells in 10 randomly selected fields of view at magnification ×200.

## 3. Results

### 3.1. Morphological Analysis

After electron irradiation, in all experimental groups (II–VII), a decrease in the area of the seminiferous tubules, their diameter, and the height of the spermatogenic epithelium compared to the samples of normal testes were found. Most of the seminiferous tubules corresponded mainly to types III, as well as types II and IV of the spermatogenic cycle.

Light microscopy of sections of the testes of the control group showed normal histoarchitectonics with physiological spermatogenesis.

In the samples of the experimental groups, a decrease in the number of germ cells, signs of degenerative changes and lysis of spermatids and spermatozoa, and a decrease in the number of spermatogonia, Sertoli and Leydig cells without changing their structure were revealed. The most serious damage to the tubules, up to aplasia of the spermatogenic epithelium and extensive vacuolization, was observed in the irradiation groups of 8–12 Gy. Destruction and wrinkling of the tubules, aplasia of the epithelium and its vacuolization progressed (Figure 1).

In the samples of the irradiation group with a dose of 2 Gy, the proportion of damaged seminiferous tubules (area—28,079.84 μm^2^; diameter—189.083 μm; *p* < 0.01) with disorganization of the epithelium and loss of cell polarity accounted for up to 1/8 of the testis (1–2 transverse sections of tubules in sight).

In the 8 Gy irradiation group, 1/3 of the seminiferous tubules showed the appearance of highly degenerated spermatids and spermatocytes combined into seminal balls (area—0.7 µm^2^; diameter—0.9 µm; *p* < 0.01). The area of single primary spermatocytes was 0.8 µm^2^ at *p* < 0.01, and the diameter was 0.9 µm at *p* < 0.01, which is 1.1 times more than normal.

The largest vacuoles were found in the seminiferous tubules of the irradiation group with a dose of 12 Gy; their area reached 3.71 µm^2^ at *p* < 0.01. The number of seminal balls doubled, and their size varied from 0.859 µm^2^ to 8 µm^2^, which is 5.0–10.0 times larger than the area of the primary spermatocyte. The dimensions of the aplastic seminiferous tubules are significantly smaller (area—14,123.72 µm^2^; diameter—76.142 µm; the height of the spermatogenic epithelium—14.2 ± 12.1 at *p* < 0.01; an increase in the number of pathological mitoses) than in the previous groups, and therefore, an increase in the interstitial component was observed testes with edema. Damage to the seminiferous tubules was 3/4.

### 3.2. Immunohistochemical Analysis

**Figure 2:**
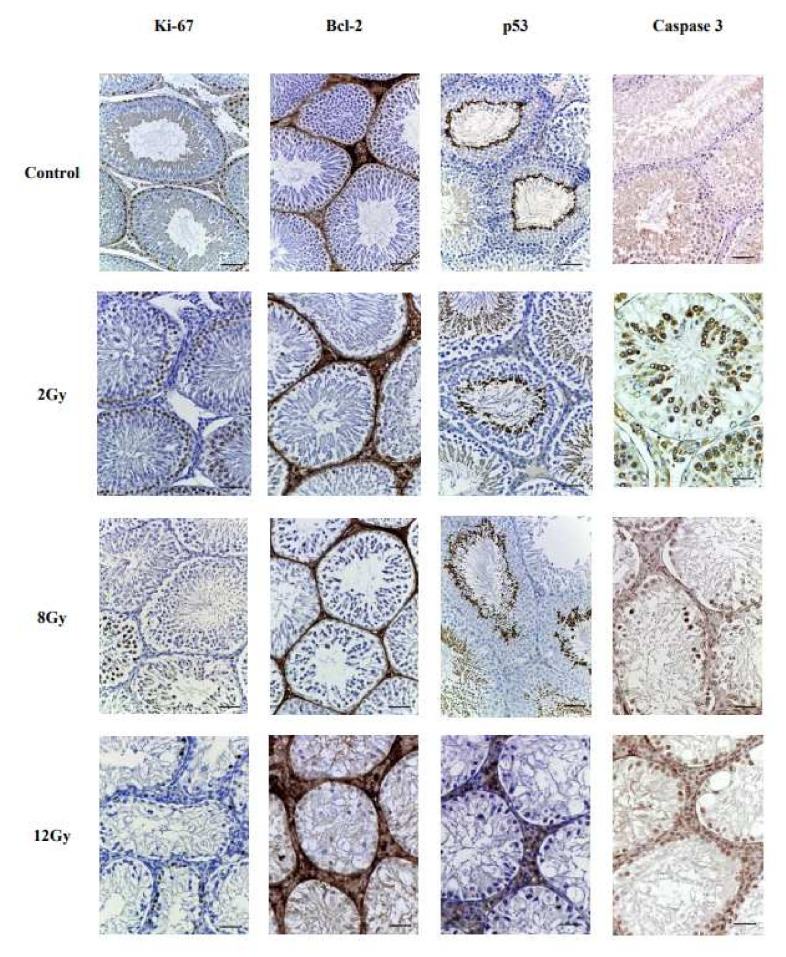
Testes of rats of experimental groups at different radiation doses, IHC staining, magn. ×200.

In all studied groups, positive IHC reactions to Ki-67, Bcl-2, p-53 and Caspase 3 were seen, but the degree of their severity correlated with the radiation dose, which indicates a disruption in the proliferative–apoptotic balance. For clarity, we present images of the seminiferous tubules’ sections of all irradiation groups (Figure 2).

Ki-67-positive reaction in spermatogonia in the 2 Gy irradiation group was 1.2 times less than in the samples of the control group, and the degree of staining tended to decrease in a dose-dependent manner. Moderate marking of gametes was observed in the samples, especially in 8 Gy and 12 Gy irradiation groups, in which the intensity of staining decreased 1.7–2.7 times compared with the control one. Primary spermatocytes showed the maximum intensity of immunolabeling. There was an increase in the number of Ki-67-positive interstitial endocrinocytes in the 12 Gy irradiation group compared with the rest groups.

The expression of apoptotic p53 in the spermatogenic epithelium of experimental groups (mainly spermatogonia and type I spermatocytes) tended to increase, though there could be seen an abrupt decline of its expression in group III. Such a decline was also noted in the expression of the Bcl-2 marker at high irradiation doses (8, 12Gy). However, a decrease in anti-apoptotic activity (Bcl-2) was still traced in experimental groups (in germ cells and in the seminal balls). A practically similar immunohistochemical pattern compared to the expression of the p53 protein was demonstrated by the expression of the anti-apoptotic protein Bcl-2: it was also noted that at high doses of irradiation, for the first time, an increase in staining of interstitial endocrinocytes was revealed compared to low doses.

The number of Cas-3-positive germ cells a week after irradiation at a dose of 2 Gy was 2.46 times higher than in the control group. Then, a constant increase in the number of Cas-3-positive germ cells was observed depending on the dose increase: when irradiated at a dose of 8 Gy—5.46 times, when irradiated at a dose of 12 Gy—6.14 times compared with the control.

## 4. Discussion

In our experimental research, we, for the first time, studied the effects of targeted electron irradiation with energy 10 MeV in different dosing modes on the spermatogenesis of male rats.

Our findings demonstrated a progressive decrease in all indicators and changes in the cells’ compound in experimental groups compared to the control one, especially while applying sublethal doses (8 and 12 Gy). According to the literature data, only premeiotic germ cells in mice’s testes, irradiated by targeted x-ray in moderate (less than 4 Gy) doses, go into apoptosis [6]. However, there are studies in which a pool of spermatids and spermatocytes, which are in postmeiotic stages, dwindles after a targeted x-ray with a dose of 2 Gy (linear accelerator «Elekta, Synergy model» [7]. Germ cells in premeiotic and postmeiotic development stages degenerate when exposed to targeted irradiation in a dose of 2 Gy (on cobalt machine «Theratron 780C, Atomic Energy of Canada Limited, Kanata, ON, Canada») [8]. Available data shows that, overall, x-rays and γ-rays (targeted and general) have the same negative effect on spermatogenesis even in low doses.

However, in our research, using a pulse electron accelerator «NOVAC-11» did not cause such morphological changes of seminiferous tubules after targeted irradiation in a dose of 2 Gy. Pathological changes were limited by the disorganization of the epithelium, decreasing the number of spermatids and spermatocytes with the preservation of their normal cell structure. The observed effect can be explained by the softer effect of electrons on testis’ tissue and impulse impact, unlike γ-radiation. In comparison with existing experimental models, the observed pathomorphological changes at the dose of 2 Gy are less destructive than after γ-radiation exposure in other scientific works, and that is why our model could be considered an optimal one for spermatogenesis study.

The rise in the dose to 8 Gy showed aggravation of aplasia. Since doses of 8 and 12 Gy showed extremely negative effects on spermatogenesis, the dose of 2 Gy could be considered borderline.

We observed the emergence of degenerated spermatocytes and spermatids, which was described by some of the authors after general γ-irradiation in doses of 8 and 10 Gy [9]. In our case, the formation of seminal balls and the dislocation of vacuolated spermatocytes in the seminiferous tubules’ gap at the same doses also indicate severe destruction of the germinal epithelium. However, somatic (Leydig and Sertoli) cells remained almost unaffected even at high, sublethal irradiation doses. This fact can be explained by their radioresistance, which has been mentioned in previous studies [6]. In a study evaluating changes in energy metabolism and testicular morphology after a single low-dose irradiation in rats, the authors concluded that only repeated exposure to irradiation can cause significant depletion of these cells [10]. Despite this specific characteristic of Sertoli and Leydig cells, the germ cells were seriously affected at 8 and 12 Gy doses, and it is possible that radiation intensity differences between γ-ray machines and weaker electron accelerators are offset after raising the dose, resulting in the same negative effect in both types of radiation. It is most likely that the 8 Gy dose is sublethal, while the 12 Gy dose is fatal for the germ cells, and condensation of heterochromatin in their nuclei is an indicator of irradiation-induced DNA damage.

Some researchers’ data show the absence of germ cells in most seminiferous tubules or the detection of only undifferentiated spermatogonia in the basal compartment after cobalt γ-irradiation in a dose of 14 Gy (Shohada-E-Tajrish machine) [11]. Practically the same morphological picture was observed in our study after irradiation with 12 Gy; however, the spermatogenic potential still persisted.

The course of physiological spermatogenesis is possible owing to the proliferation-apoptotic gametes’ balance. In our study, the IHC reactions confirmed that the level of proapoptotic p53 marker increases, while the level of the proliferation Ki-67 and antia-poptotic Bcl-2 markers decreases after germ cells’ damage, and these results agree with previous studies that show similar results unless the cells are radioresistant [12,13,14]. At the same time, an abrupt decrease in expression of Bcl-2 and p53 at high doses of exposure can be explained by a critically low number of remaining living cells. Expanding involvement of p53 indicates the apoptosis of a greater amount of germ cells, especially spermatogonia, which showed the most intensive staining at an 8 Gy dose. The role of p53 overexpression after cell irradiation was also proved in other studies where x-rays were used [15]. This fact indicates that the increased activity of p53 is a response to various types of radiation, including electron irradiation.

Moreover, in our previous study, the results of the TUNEL reaction and the spectrum of caspase (−3, −8, −9) indicate the internal (mitochondrial) pathway of apoptosis of the germ cells of irradiated testes and a more pronounced increase in the expression of caspase-9 in the group with the highest radiation exposure (8 Gy)—on a possible correlation between the absorbed dose of ionizing radiation and the degree of damage to mitochondria [16].

Thus, we can assume that the effects of irradiation and the damage level of spermatogenic epithelium depend on doses. Probably, a threshold dose is much higher for electron irradiation on the accelerator “NOVAC-11” due to a lighter dosing mode. Another important factor of irradiation efficiency is its type (β-irradiation (electron), γ-rays, x-rays etc.), its focus (general, targeted), activity and power of the machine and spermatogenesis stage. This assumption was confirmed by the results of the morphometrical analysis: decreasing of testes mass, seminiferous tubules diameter and height of the germinal epithelium due to a reduction in the number of differentiated germ cells, which depends on the type of radiational exposure at different dosing regimens [17,18].

## 5. Conclusions

The detected progressive pathomorphological changes of histoarchitectonics in testes are dose-dependent. These alterations manifest in a decrease in the number of spermatogonia and other germ cells and are already seen on the seventh day after irradiation. The basis of the revealed hyperplasia of Leydig cells as a reaction to irradiation is a sharp imbalance of pro- and anti-apoptotic factors toward dominance in most cells of the expression of the anti-apoptotic factor Bcl-2 (with less significant activation of Ki-67 expression) and extremely low expression of p53. With an increase in the radiation dose, the number of apoptotic bodies also increases. In all experimental groups, a dose-dependent decrease in Ki-67 positive and Bcl-2 positive germ cells was noted a week after irradiation and, on the contrary, an increase in p53-positive and Caspase-3 positive germ cells. The recorded morphological changes of spermatogenesis when exposed to the pulsed electron irradiation on the accelerator “NOVAC-11” are more gentle, in contrast to other applied machines.

## Figures and Tables

**Table 1:** Comparative characteristics of IHC-positive cells in seminiferous tubules after electron irradiation (%), *p* < 0.01.

Radiation Dose, Gy	Ki-67	Bcl-2	p53	Caspase 3
Control	76.0 ± 3.7	53.4 ± 2.6	54.3 ± 2.7	5.0 ± 0.2
2	53.7 ± 2.6	29.3 ± 1.3	57.1 ± 2.8	12.3 ± 0.5
8	25.3 ± 1.1	1.0 ± 0.1	68.5 ± 3.3	27.3 ± 1.2
12	11.0 ± 0.1	0.4 ± 0.1	76.8 ± 3.6	30.7 ± 1.5

## Data Availability

All data and materials, as well as software application or custom code, support their published claims, comply with field standards, and are openly available.
